# SERVICE LEARNING TO PREPARE OCCUPATIONAL THERAPY STUDENTS TO MEET THE NEEDS OF VETERANS WITH LATE-STAGE DEMENTIA

**DOI:** 10.1093/geroni/igae098.1521

**Published:** 2024-12-31

**Authors:** Scott Trudeau

**Affiliations:** American Occupational Therapy Association, Stow, Massachusetts, United States

## Abstract

One of the challenges to providing activity programs in dementia units is meeting a wide range of residents’ needs. Programs are typically directed to the more moderately functional, often not meeting the needs of the least and most functional. The recognition that a significant cohort (approximately 20%) of residents on a dementia unit at Veterans Health Administration were typically excluded from group activities was of concern. This cohort came to be referred to as “VIPs” (Very Isolated Patients). The reason for their isolation was difficulty tailoring activities to their limited function within a group context. While more individualized approaches would be ideal, limited staffing precluded this approach. Developing a structured intervention that could be deployed by students to this vulnerable population seemed advantageous. The Reading Buddies Program was developed in which students were paired with a VIP resident with the assignment to meet with them weekly to engage in reading for a 45-minute session. Students read to residents in pairs, with one student facilitating and another student observing and gathering data. Data was collected in the form of qualitative student reflections and the Observed Measure of Engagement (OME). Students reported many opportunities for growth and mastery. The OME data reflected continued capacity for resident engagement in reading activities. Findings support the conclusion that by providing cognitive stimulation and social interaction for residents with later stage dementia, The Reading Buddies Program provided hands-on experience for occupational therapy graduate students to address the challenging functional and interpersonal needs of this cohort.

